# Motives and Goals for Sports and Exercise Therapy during Treatment of Depression

**DOI:** 10.1192/j.eurpsy.2025.1321

**Published:** 2025-08-26

**Authors:** K. Friedrich, C. A. Penkov, J. Krieger, V. Rößner-Ruff, M. Wendt, M. Ziegenbein

**Affiliations:** 1 Forschung & Entwicklung; 2 Sporttherapie, Wahrendorff Klinikum, Sehnde, Germany

## Abstract

**Introduction:**

A growing body of evidence suggests that physical activity can be an effective treatment for depression. In consideration of individual conditions, Sports and Exercise Therapy may be used as standalone or complementary treatment during partial or full-time inpatient treatment. However, current data indicate that only a minority of patients make use of it during the course of their treatment. The beneficial health effects of exercise on mental health can only be realized if the exercise is actually undertaken. Therefore, further research is required on the motivational psychological aspects of participation in Sports and Exercise Therapy. It can be assumed that it is crucial to consider the individual patient preferences when initiating and sustaining physical activity. Moreover, there are notable differences between men and women in terms of their motivation for engaging in exercise.

**Objectives:**

The present study examines gender-based differences in the motivation underlying the participation in Sports and Exercise Therapy during the course of inpatient treatment for depression, whether on a partial or full-time basis.

**Methods:**

In a psychotherapeutic and psychosomatic hospital, motives for Sports and Exercise Therapy of female and male (age 35-64) patients with a primary diagnosis of depression were recorded using the Bernese Motive and Goal Inventory in Leisure and Health Sports. The motives analysed include contact, body/appearance, competition/performance, distraction/catharsis, health, fitness and aesthetics. The survey was conducted within seven days of admission to either partial or full-time inpatient treatment setting.

**Results:**

The total number of patients included in the analysis was 140, comprising 65.0% male (mean-age=48.0, 21.0% full-time treatment) and 35.0% female (mean-age=50.7, 43.0% full-time treatment). The most important motives for both men and women are health (M male=4.29, M female=4.37), fitness (M male=4.20, M female=4.20) and distraction/catharsis (M male=3.72, M female=3.80) due to the highest mean scores. Compared to women (M=1,60, SD=0.77) men (M=2.19, SD=1.93) reported significantly higher mean score for the competition/performance motive (U-test, z=3.987, p<.001). No further significant gender differences were identified.

**Conclusions:**

Significant gender differences were found only for the motive competition/ performance, despite this motive rated lowest on average. The absence of further gender differences may be due to the fact that gender differences may be of less importance for Sports and Exercise Therapy in the treatment context. On average health, fitness, and distraction/ catharsis were rated highest by both genders. This is consistent with typical symptoms during depression, which involve distraction from negative thoughts as well as recovering physical fitness to cope with everyday life. All authors of this study declare that they have no conflicts of interest.

**Disclosure of Interest:**

None Declared

